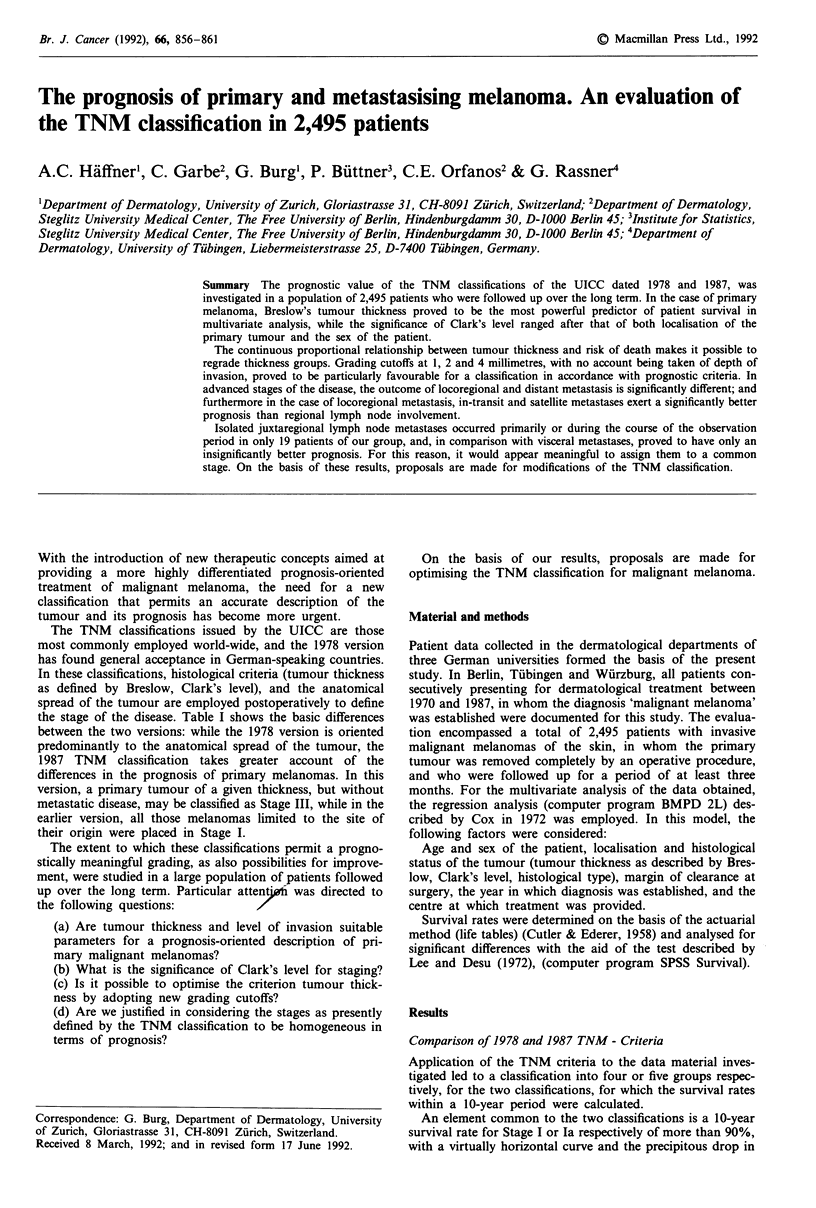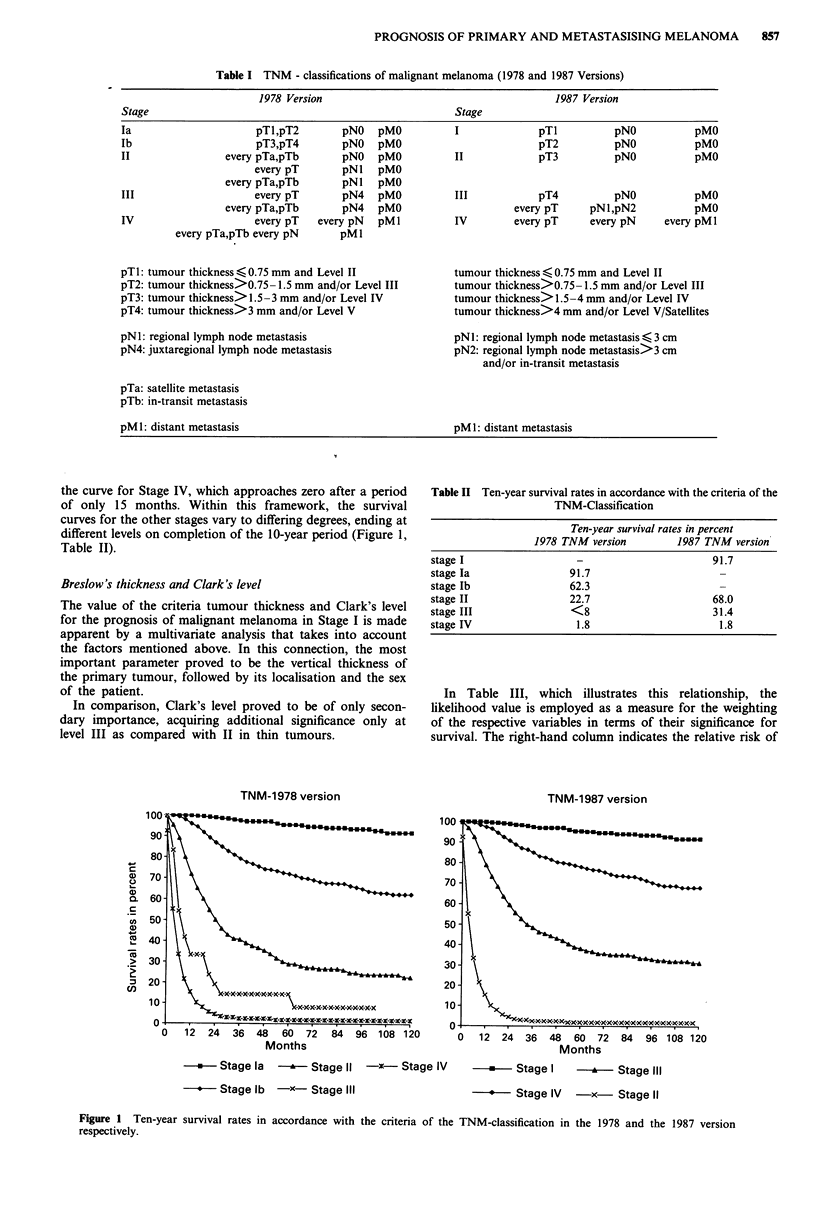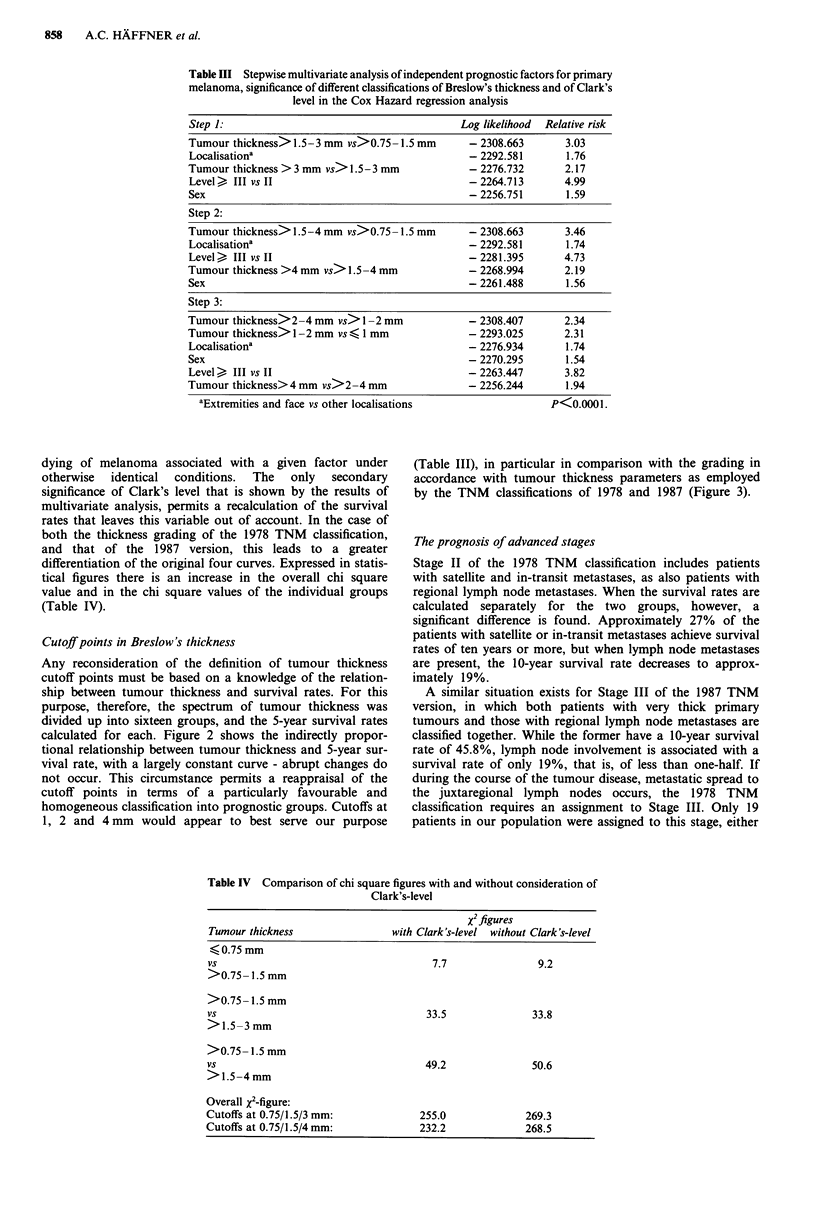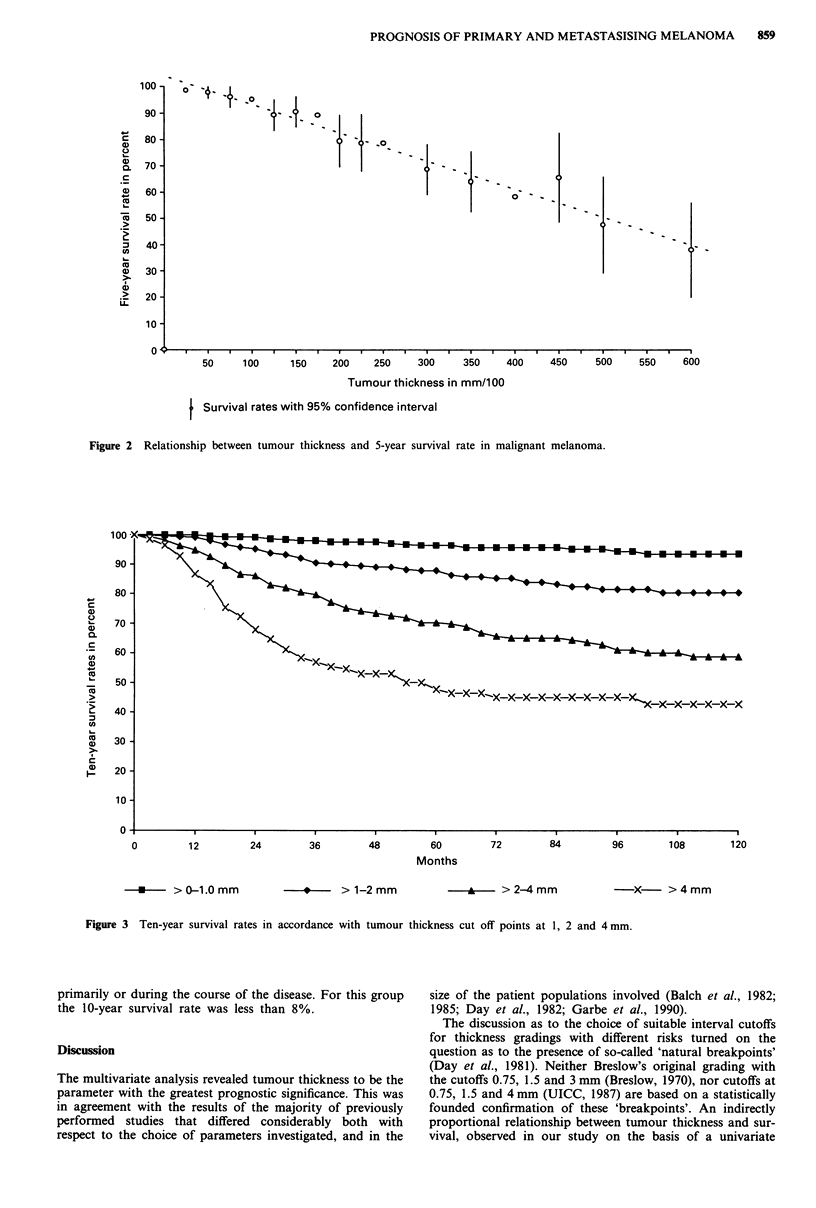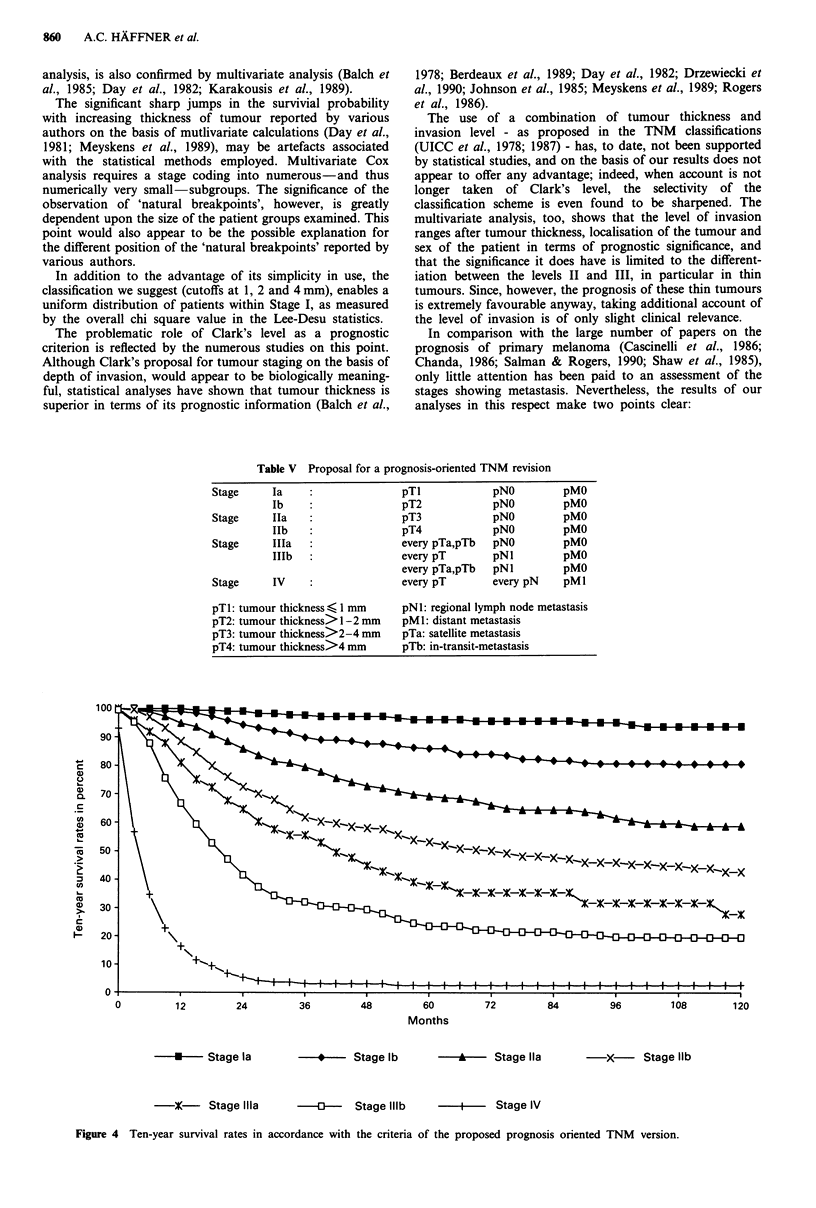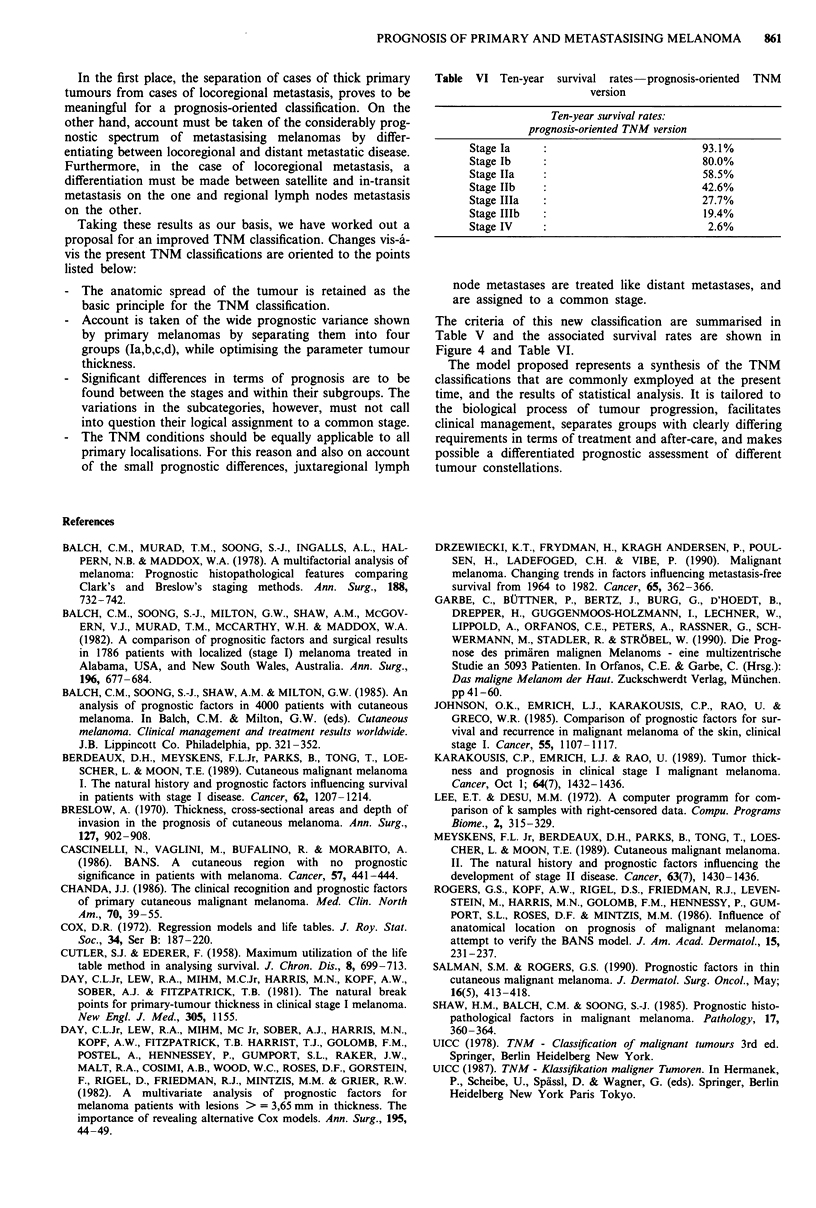# The prognosis of primary and metastasising melanoma. An evaluation of the TNM classification in 2,495 patients.

**DOI:** 10.1038/bjc.1992.373

**Published:** 1992-11

**Authors:** A. C. Häffner, C. Garbe, G. Burg, P. Büttner, C. E. Orfanos, G. Rassner

**Affiliations:** Department of Dermatology, University of Zurich, Switzerland.

## Abstract

The prognostic value of the TNM classifications of the UICC dated 1978 and 1987, was investigated in a population of 2,495 patients who were followed up over the long term. In the case of primary melanoma, Breslow's tumour thickness proved to be the most powerful predictor of patient survival in multivariate analysis, while the significance of Clark's level ranged after that of both localisation of the primary tumour and the sex of the patient. The continuous proportional relationship between tumour thickness and risk of death makes it possible to regrade thickness groups. Grading cutoffs at 1, 2 and 4 millimetres, with no account being taken of depth of invasion, proved to be particularly favourable for a classification in accordance with prognostic criteria. In advanced stages of the disease, the outcome of locoregional and distant metastasis is significantly different; and furthermore in the case of locoregional metastasis, in-transit and satellite metastases exert a significantly better prognosis than regional lymph node involvement. Isolated juxtaregional lymph node metastases occurred primarily or during the course of the observation period in only 19 patients of our group, and, in comparison with visceral metastases, proved to have only an insignificantly better prognosis. For this reason, it would appear meaningful to assign them to a common stage. On the basis of these results, proposals are made for modifications of the TNM classification.


*Br. J. Cancer* (1992), **66**, 856–861 © Macmillan Press Ltd., 1992

### The prognosis of primary and metastasising melanoma. An evaluation of the TNM classification in 2,495 patients

A.C. Häffner[1], C. Garbe[2], G. Burg[1], P. Büttner[3], C.E. Orfanos[2] & G. Rassner[4]

[1]*Department of Dermatology, University of Zurich, Gloriastrasse 31, CH-8091 Zürich, Switzerland;* [2]*Department of Dermatology, Steglitz University Medical Center, The Free University of Berlin, Hindenburgdamm 30, D-1000 Berlin 45;* [3]*Institute for Statistics, Steglitz University Medical Center, The Free University of Berlin, Hindenburgdamm 30, D-1000 Berlin 45;* [4]*Department of Dermatology, University of Tübingen, Liebermeisterstrasse 25, D-7400 Tübingen, Germany.*

**Summary** The prognostic value of the TNM classifications of the UICC dated 1978 and 1987, was investigated in a population of 2,495 patients who were followed up over the long term. In the case of primary melanoma, Breslow's tumour thickness proved to be the most powerful predictor of patient survival in multivariate analysis, while the significance of Clark's level ranged after that of both localisation of the primary tumour and the sex of the patient.

The continuous proportional relationship between tumour thickness and risk of death makes it possible to regrade thickness groups. Grading cutoffs at 1, 2 and 4 millimetres, with no account being taken of depth of invasion, proved to be particularly favourable for a classification in accordance with prognostic criteria. In advanced stages of the disease, the outcome of locoregional and distant metastasis is significantly different; and furthermore in the case of locoregional metastasis, in-transit and satellite metastases exert a significantly better prognosis than regional lymph node involvement.

Isolated juxtaregional lymph node metastases occurred primarily or during the course of the observation period in only 19 patients of our group, and, in comparison with visceral metastases, proved to have only an insignificantly better prognosis. For this reason, it would appear meaningful to assign them to a common stage. On the basis of these results, proposals are made for modifications of the TNM classification.

With the introduction of new therapeutic concepts aimed at providing a more highly differentiated prognosis-oriented treatment of malignant melanoma, the need for a new classification that permits an accurate description of the tumour and its prognosis has become more urgent.

The TNM classifications issued by the UICC are those most commonly employed world-wide, and the 1978 version has found general acceptance in German-speaking countries. In these classifications, histological criteria (tumour thickness as defined by Breslow, Clark's level), and the anatomical spread of the tumour are employed postoperatively to define the stage of the disease. Table I shows the basic differences between the two versions: while the 1978 version is oriented predominantly to the anatomical spread of the tumour, the 1987 TNM classification takes greater account of the differences in the prognosis of primary melanomas. In this version, a primary tumour of a given thickness, but without metastatic disease, may be classified as Stage III, while in the earlier version, all those melanomas limited to the site of their origin were placed in Stage I.

The extent to which these classifications permit a prognostically meaningful grading, as also possibilities for improvement, were studied in a large population of patients followed up over the long term. Particular attention was directed to the following questions:

(a) Are tumour thickness and level of invasion suitable parameters for a prognosis-oriented description of primary malignant melanomas?
(b) What is the significance of Clark's level for staging?
(c) Is it possible to optimise the criterion tumour thickness by adopting new grading cutoffs?
(d) Are we justified in considering the stages as presently defined by the TNM classification to be homogeneous in terms of prognosis?

Correspondence: G. Burg, Department of Dermatology, University of Zurich, Gloriastrasse 31, CH-8091 Zürich, Switzerland.
Received 8 March, 1992; and in revised form 17 June 1992.

On the basis of our results, proposals are made for optimising the TNM classification for malignant melanoma.

### Material and methods

Patient data collected in the dermatological departments of three German universities formed the basis of the present study. In Berlin, Tübingen and Würzburg, all patients consecutively presenting for dermatological treatment between 1970 and 1987, in whom the diagnosis 'malignant melanoma' was established were documented for this study. The evaluation encompassed a total of 2,495 patients with invasive malignant melanomas of the skin, in whom the primary tumour was removed completely by an operative procedure, and who were followed up for a period of at least three months. For the multivariate analysis of the data obtained, the regression analysis (computer program BMPD 2L) described by Cox in 1972 was employed. In this model, the following factors were considered:

Age and sex of the patient, localisation and histological status of the tumour (tumour thickness as described by Breslow, Clark's level, histological type), margin of clearance at surgery, the year in which diagnosis was established, and the centre at which treatment was provided.

Survival rates were determined on the basis of the actuarial method (life tables) (Cutler & Ederer, 1958) and analysed for significant differences with the aid of the test described by Lee and Desu (1972), (computer program SPSS Survival).

### Results

### *Comparison of 1978 and 1987 TNM - Criteria*

Application of the TNM criteria to the data material investigated led to a classification into four or five groups respectively, for the two classifications, for which the survival rates within a 10-year period were calculated.

An element common to the two classifications is a 10-year survival rate for Stage I or Ia respectively of more than 90%, with a virtually horizontal curve and the precipitous drop in

**Table I** TNM - classifications of malignant melanoma (1978 and 1987 Versions)

| *Stage* | *1978 Version* | | | *Stage* | *1987 Version* | | |
|---|---|---|---|---|---|---|---|
| Ia | pT1,pT2 | pN0 | pM0 | I | pT1 | pN0 | pM0 |
| Ib | pT3,pT4 | pN0 | pM0 | | pT2 | pN0 | pM0 |
| II | every pTa,pTb | pN0 | pM0 | II | pT3 | pN0 | pM0 |
| | every pT | pN1 | pM0 | | | | |
| | every pTa,pTb | pN1 | pM0 | | | | |
| III | every pT | pN4 | pM0 | III | pT4 | pN0 | pM0 |
| | every pTa,pTb | pN4 | pM0 | | every pT | pN1,pN2 | pM0 |
| IV | every pT | every pN | pM1 | IV | every pT | every pN | every pM1 |
| | every pTa,pTb every pN | pM1 | | | | | |

| 1978 Version | 1987 Version |
|---|---|
| pT1: tumour thickness ≤ 0.75 mm and Level II | tumour thickness ≤ 0.75 mm and Level II |
| pT2: tumour thickness > 0.75–1.5 mm and/or Level III | tumour thickness > 0.75–1.5 mm and/or Level III |
| pT3: tumour thickness > 1.5–3 mm and/or Level IV | tumour thickness > 1.5–4 mm and/or Level IV |
| pT4: tumour thickness > 3 mm and/or Level V | tumour thickness > 4 mm and/or Level V/Satellites |
| pN1: regional lymph node metastasis | pN1: regional lymph node metastasis ≤ 3 cm |
| pN4: juxtaregional lymph node metastasis | pN2: regional lymph node metastasis > 3 cm and/or in-transit metastasis |
| pTa: satellite metastasis | |
| pTb: in-transit metastasis | |
| pM1: distant metastasis | pM1: distant metastasis |

the curve for Stage IV, which approaches zero after a period of only 15 months. Within this framework, the survival curves for the other stages vary to differing degrees, ending at different levels on completion of the 10-year period (Figure 1, Table II).

### *Breslow's thickness and Clark's level*

The value of the criteria tumour thickness and Clark's level for the prognosis of malignant melanoma in Stage I is made apparent by a multivariate analysis that takes into account the factors mentioned above. In this connection, the most important parameter proved to be the vertical thickness of the primary tumour, followed by its localisation and the sex of the patient.

In comparison, Clark's level proved to be of only secondary importance, acquiring additional significance only at level III as compared with II in thin tumours.

**Table II** Ten-year survival rates in accordance with the criteria of the TNM-Classification

| | *Ten-year survival rates in percent* | |
|---|---|---|
| | *1978 TNM version* | *1987 TNM version* |
| stage I | – | 91.7 |
| stage Ia | 91.7 | – |
| stage Ib | 62.3 | – |
| stage II | 22.7 | 68.0 |
| stage III | <8 | 31.4 |
| stage IV | 1.8 | 1.8 |

In Table III, which illustrates this relationship, the likelihood value is employed as a measure for the weighting of the respective variables in terms of their significance for survival. The right-hand column indicates the relative risk of

**Figure 1** Ten-year survival rates in accordance with the criteria of the TNM-classification in the 1978 and the 1987 version respectively.

**Table III** Stepwise multivariate analysis of independent prognostic factors for primary melanoma, significance of different classifications of Breslow's thickness and of Clark's level in the Cox Hazard regression analysis

| *Step 1:* | *Log likelihood* | *Relative risk* |
|---|---|---|
| Tumour thickness>1.5–3 mm *vs*>0.75–1.5 mm | – 2308.663 | 3.03 |
| Localisation[a] | – 2292.581 | 1.76 |
| Tumour thickness >3 mm *vs*>1.5–3 mm | – 2276.732 | 2.17 |
| Level ≥ III *vs* II | – 2264.713 | 4.99 |
| Sex | – 2256.751 | 1.59 |
| Step 2: | | |
| Tumour thickness>1.5–4 mm *vs*>0.75–1.5 mm | – 2308.663 | 3.46 |
| Localisation[a] | – 2292.581 | 1.74 |
| Level ≥ III *vs* II | – 2281.395 | 4.73 |
| Tumour thickness >4 mm *vs*>1.5–4 mm | – 2268.994 | 2.19 |
| Sex | – 2261.488 | 1.56 |
| Step 3: | | |
| Tumour thickness>2–4 mm *vs*>1–2 mm | – 2308.407 | 2.34 |
| Tumour thickness>1–2 mm *vs*≤1 mm | – 2293.025 | 2.31 |
| Localisation[a] | – 2276.934 | 1.74 |
| Sex | – 2270.295 | 1.54 |
| Level ≥ III *vs* II | – 2263.447 | 3.82 |
| Tumour thickness>4 mm *vs*>2–4 mm | – 2256.244 | 1.94 |

[a]Extremities and face *vs* other localisations $P < 0.0001$.

dying of melanoma associated with a given factor under otherwise identical conditions. The only secondary significance of Clark's level that is shown by the results of multivariate analysis, permits a recalculation of the survival rates that leaves this variable out of account. In the case of both the thickness grading of the 1978 TNM classification, and that of the 1987 version, this leads to a greater differentiation of the original four curves. Expressed in statistical figures there is an increase in the overall chi square value and in the chi square values of the individual groups (Table IV).

### *Cutoff points in Breslow's thickness*

Any reconsideration of the definition of tumour thickness cutoff points must be based on a knowledge of the relationship between tumour thickness and survival rates. For this purpose, therefore, the spectrum of tumour thickness was divided up into sixteen groups, and the 5-year survival rates calculated for each. Figure 2 shows the indirectly proportional relationship between tumour thickness and 5-year survival rate, with a largely constant curve - abrupt changes do not occur. This circumstance permits a reappraisal of the cutoff points in terms of a particularly favourable and homogeneous classification into prognostic groups. Cutoffs at 1, 2 and 4 mm would appear to best serve our purpose (Table III), in particular in comparison with the grading in accordance with tumour thickness parameters as employed by the TNM classifications of 1978 and 1987 (Figure 3).

### *The prognosis of advanced stages*

Stage II of the 1978 TNM classification includes patients with satellite and in-transit metastases, as also patients with regional lymph node metastases. When the survival rates are calculated separately for the two groups, however, a significant difference is found. Approximately 27% of the patients with satellite or in-transit metastases achieve survival rates of ten years or more, but when lymph node metastases are present, the 10-year survival rate decreases to approximately 19%.

A similar situation exists for Stage III of the 1987 TNM version, in which both patients with very thick primary tumours and those with regional lymph node metastases are classified together. While the former have a 10-year survival rate of 45.8%, lymph node involvement is associated with a survival rate of only 19%, that is, of less than one-half. If during the course of the tumour disease, metastatic spread to the juxtaregional lymph nodes occurs, the 1978 TNM classification requires an assignment to Stage III. Only 19 patients in our population were assigned to this stage, either

**Table IV** Comparison of chi square figures with and without consideration of Clark's-level

| *Tumour thickness* | $\chi^2$ *figures* *with Clark's-level* | *without Clark's-level* |
|---|---|---|
| ≤0.75 mm *vs* >0.75–1.5 mm | 7.7 | 9.2 |
| >0.75–1.5 mm *vs* >1.5–3 mm | 33.5 | 33.8 |
| >0.75–1.5 mm *vs* >1.5–4 mm | 49.2 | 50.6 |
| Overall $\chi^2$-figure: | | |
| Cutoffs at 0.75/1.5/3 mm: | 255.0 | 269.3 |
| Cutoffs at 0.75/1.5/4 mm: | 232.2 | 268.5 |

**Figure 2** Relationship between tumour thickness and 5-year survival rate in malignant melanoma.

**Figure 3** Ten-year survival rates in accordance with tumour thickness cut off points at 1, 2 and 4 mm.

primarily or during the course of the disease. For this group the 10-year survival rate was less than 8%.

### Discussion

The multivariate analysis revealed tumour thickness to be the parameter with the greatest prognostic significance. This was in agreement with the results of the majority of previously performed studies that differed considerably both with respect to the choice of parameters investigated, and in the size of the patient populations involved (Balch *et al.*, 1982; 1985; Day *et al.*, 1982; Garbe *et al.*, 1990).

The discussion as to the choice of suitable interval cutoffs for thickness gradings with different risks turned on the question as to the presence of so-called 'natural breakpoints' (Day *et al.*, 1981). Neither Breslow's original grading with the cutoffs 0.75, 1.5 and 3 mm (Breslow, 1970), nor cutoffs at 0.75, 1.5 and 4 mm (UICC, 1987) are based on a statistically founded confirmation of these 'breakpoints'. An indirectly proportional relationship between tumour thickness and survival, observed in our study on the basis of a univariate analysis, is also confirmed by multivariate analysis (Balch *et al.*, 1985; Day *et al.*, 1982; Karakousis *et al.*, 1989).

The significant sharp jumps in the survivial probability with increasing thickness of tumour reported by various authors on the basis of mutlivariate calculations (Day *et al.*, 1981; Meyskens *et al.*, 1989), may be artefacts associated with the statistical methods employed. Multivariate Cox analysis requires a stage coding into numerous—and thus numerically very small—subgroups. The significance of the observation of 'natural breakpoints', however, is greatly dependent upon the size of the patient groups examined. This point would also appear to be the possible explanation for the different position of the 'natural breakpoints' reported by various authors.

In addition to the advantage of its simplicity in use, the classification we suggest (cutoffs at 1, 2 and 4 mm), enables a uniform distribution of patients within Stage I, as measured by the overall chi square value in the Lee-Desu statistics.

The problematic role of Clark's level as a prognostic criterion is reflected by the numerous studies on this point. Although Clark's proposal for tumour staging on the basis of depth of invasion, would appear to be biologically meaningful, statistical analyses have shown that tumour thickness is superior in terms of its prognostic information (Balch *et al.*, 1978; Berdeaux *et al.*, 1989; Day *et al.*, 1982; Drzewiecki *et al.*, 1990; Johnson *et al.*, 1985; Meyskens *et al.*, 1989; Rogers *et al.*, 1986).

The use of a combination of tumour thickness and invasion level - as proposed in the TNM classifications (UICC *et al.*, 1978; 1987) - has, to date, not been supported by statistical studies, and on the basis of our results does not appear to offer any advantage; indeed, when account is not longer taken of Clark's level, the selectivity of the classification scheme is even found to be sharpened. The multivariate analysis, too, shows that the level of invasion ranges after tumour thickness, localisation of the tumour and sex of the patient in terms of prognostic significance, and that the significance it does have is limited to the differentiation between the levels II and III, in particular in thin tumours. Since, however, the prognosis of these thin tumours is extremely favourable anyway, taking additional account of the level of invasion is of only slight clinical relevance.

In comparison with the large number of papers on the prognosis of primary melanoma (Cascinelli *et al.*, 1986; Chanda, 1986; Salman & Rogers, 1990; Shaw *et al.*, 1985), only little attention has been paid to an assessment of the stages showing metastasis. Nevertheless, the results of our analyses in this respect make two points clear:

**Table V** Proposal for a prognosis-oriented TNM revision

| | | | | | |
|---|---|---|---|---|---|
| Stage | Ia | : | pT1 | pN0 | pM0 |
| | Ib | : | pT2 | pN0 | pM0 |
| Stage | IIa | : | pT3 | pN0 | pM0 |
| | IIb | : | pT4 | pN0 | pM0 |
| Stage | IIIa | : | every pTa,pTb | pN0 | pM0 |
| | IIIb | : | every pT | pN1 | pM0 |
| | | | every pTa,pTb | pN1 | pM0 |
| Stage | IV | : | every pT | every pN | pM1 |

pT1: tumour thickness ≤ 1 mm pT2: tumour thickness > 1–2 mm pT3: tumour thickness > 2–4 mm pT4: tumour thickness > 4 mm pN1: regional lymph node metastasis
pM1: distant metastasis
pTa: satellite metastasis
pTb: in-transit-metastasis

**Figure 4** Ten-year survival rates in accordance with the criteria of the proposed prognosis oriented TNM version.

In the first place, the separation of cases of thick primary tumours from cases of locoregional metastasis, proves to be meaningful for a prognosis-oriented classification. On the other hand, account must be taken of the considerably prognostic spectrum of metastasising melanomas by differentiating between locoregional and distant metastatic disease. Furthermore, in the case of locoregional metastasis, a differentiation must be made between satellite and in-transit metastasis on the one and regional lymph nodes metastasis on the other.

Taking these results as our basis, we have worked out a proposal for an improved TNM classification. Changes vis-á-vis the present TNM classifications are oriented to the points listed below:

- The anatomic spread of the tumour is retained as the basic principle for the TNM classification.
- Account is taken of the wide prognostic variance shown by primary melanomas by separating them into four groups (Ia,b,c,d), while optimising the parameter tumour thickness.
- Significant differences in terms of prognosis are to be found between the stages and within their subgroups. The variations in the subcategories, however, must not call into question their logical assignment to a common stage.
- The TNM conditions should be equally applicable to all primary localisations. For this reason and also on account of the small prognostic differences, juxtaregional lymph node metastases are treated like distant metastases, and are assigned to a common stage.

**Table VI** Ten-year survival rates—prognosis-oriented TNM version

| *Ten-year survival rates: prognosis-oriented TNM version* | | |
|---|---|---|
| Stage Ia | : | 93.1% |
| Stage Ib | : | 80.0% |
| Stage IIa | : | 58.5% |
| Stage IIb | : | 42.6% |
| Stage IIIa | : | 27.7% |
| Stage IIIb | : | 19.4% |
| Stage IV | : | 2.6% |

The criteria of this new classification are summarised in Table V and the associated survival rates are shown in Figure 4 and Table VI.

The model proposed represents a synthesis of the TNM classifications that are commonly exmployed at the present time, and the results of statistical analysis. It is tailored to the biological process of tumour progression, facilitates clinical management, separates groups with clearly differing requirements in terms of treatment and after-care, and makes possible a differentiated prognostic assessment of different tumour constellations.

### References

BALCH, C.M., MURAD, T.M., SOONG, S.-J., INGALLS, A.L., HALPERN, N.B. & MADDOX, W.A. (1978). A multifactorial analysis of melanoma: Prognostic histopathological features comparing Clark's and Breslow's staging methods. *Ann. Surg.*, **188**, 732–742.

BALCH, C.M., SOONG, S.-J., MILTON, G.W., SHAW, A.M., McGOVERN, V.J., MURAD, T.M., McCARTHY, W.H. & MADDOX, W.A. (1982). A comparison of prognositic factors and surgical results in 1786 patients with localized (stage I) melanoma treated in Alabama, USA, and New South Wales, Australia. *Ann. Surg.*, **196**, 677–684.

BALCH, C.M., SOONG, S.-J., SHAW, A.M. & MILTON, G.W. (1985). An analysis of prognostic factors in 4000 patients with cutaneous melanoma. In Balch, C.M. & Milton, G.W. (eds). *Cutaneous melanoma. Clinical management and treatment results worldwide.* J.B. Lippincott Co. Philadelphia, pp. 321–352.

BERDEAUX, D.H., MEYSKENS, F.L.Jr, PARKS, B., TONG, T., LOESCHER, L. & MOON, T.E. (1989). Cutaneous malignant melanoma I. The natural history and prognostic factors influencing survival in patients with stage I disease. *Cancer*, **62**, 1207–1214.

BRESLOW, A. (1970). Thickness, cross-sectional areas and depth of invasion in the prognosis of cutaneous melanoma. *Ann. Surg.*, **127**, 902–908.

CASCINELLI, N., VAGLINI, M., BUFALINO, R. & MORABITO, A. (1986). BANS. A cutaneous region with no prognostic significance in patients with melanoma. *Cancer*, **57**, 441–444.

CHANDA, J.J. (1986). The clinical recognition and prognostic factors of primary cutaneous malignant melanoma. *Med. Clin. North Am.*, **70**, 39–55.

COX, D.R. (1972). Regression models and life tables. *J. Roy. Stat. Soc.*, **34**, Ser B: 187–220.

CUTLER, S.J. & EDERER, F. (1958). Maximum utilization of the life table method in analysing survival. *J. Chron. Dis.*, **8**, 699–713.

DAY, C.L.Jr, LEW, R.A., MIHM, M.C.Jr, HARRIS, M.N., KOPF, A.W., SOBER, A.J. & FITZPATRICK, T.B. (1981). The natural break points for primary-tumour thickness in clinical stage I melanoma. *New Engl. J. Med.*, **305**, 1155.

DAY, C.L.Jr, LEW, R.A., MIHM, MC Jr, SOBER, A.J., HARRIS, M.N., KOPF, A.W., FITZPATRICK, T.B. HARRIST, T.J., GOLOMB, F.M., POSTEL, A., HENNESSEY, P., GUMPORT, S.L., RAKER, J.W., MALT, R.A., COSIMI, A.B., WOOD, W.C., ROSES, D.F., GORSTEIN, F., RIGEL, D., FRIEDMAN, R.J., MINTZIS, M.M. & GRIER, R.W. (1982). A multivariate analysis of prognostic factors for melanoma patients with lesions >= 3,65 mm in thickness. The importance of revealing alternative Cox models. *Ann. Surg.*, **195**, 44–49.

DRZEWIECKI, K.T., FRYDMAN, H., KRAGH ANDERSEN, P., POULSEN, H., LADEFOGED, C.H. & VIBE, P. (1990). Malignant melanoma. Changing trends in factors influencing metastasis-free survival from 1964 to 1982. *Cancer*, **65**, 362–366.

GARBE, C., BÜTTNER, P., BERTZ, J., BURG, G., D'HOEDT, B., DREPPER, H., GUGGENMOOS-HOLZMANN, I., LECHNER, W., LIPPOLD, A., ORFANOS, C.E., PETERS, A., RASSNER, G., SCHWERMANN, M., STADLER, R. & STRÖBEL, W. (1990). Die Prognose des primären malignen Melanoms - eine multizentrische Studie an 5093 Patienten. In Orfanos, C.E. & Garbe, C. (Hrsg.): *Das maligne Melanom der Haut.* Zuckschwerdt Verlag, München. pp 41–60.

JOHNSON, O.K., EMRICH, L.J., KARAKOUSIS, C.P., RAO, U. & GRECO, W.R. (1985). Comparison of prognostic factors for survival and recurrence in malignant melanoma of the skin, clinical stage I. *Cancer*, **55**, 1107–1117.

KARAKOUSIS, C.P., EMRICH, L.J. & RAO, U. (1989). Tumor thickness and prognosis in clinical stage I malignant melanoma. *Cancer*, Oct 1; **64**(7), 1432–1436.

LEE, E.T. & DESU, M.M. (1972). A computer programm for comparison of k samples with right-censored data. *Compu. Programs Biome.*, **2**, 315–329.

MEYSKENS, F.L. Jr, BERDEAUX, D.H., PARKS, B., TONG, T., LOESCHER, L. & MOON, T.E. (1989). Cutaneous malignant melanoma. II. The natural history and prognostic factors influencing the development of stage II disease. *Cancer*, **63**(7), 1430–1436.

ROGERS, G.S., KOPF, A.W., RIGEL, D.S., FRIEDMAN, R.J., LEVENSTEIN, M., HARRIS, M.N., GOLOMB, F.M., HENNESSY, P., GUMPORT, S.L., ROSES, D.F. & MINTZIS, M.M. (1986). Influence of anatomical location on prognosis of malignant melanoma: attempt to verify the BANS model. *J. Am. Acad. Dermatol.*, **15**, 231–237.

SALMAN, S.M. & ROGERS, G.S. (1990). Prognostic factors in thin cutaneous malignant melanoma. *J. Dermatol. Surg. Oncol.*, May; **16**(5), 413–418.

SHAW, H.M., BALCH, C.M. & SOONG, S.-J. (1985). Prognostic histopathological factors in malignant melanoma. *Pathology*, **17**, 360–364.

UICC (1978). *TNM - Classification of malignant tumours* 3rd ed. Springer, Berlin Heidelberg New York.

UICC (1987). *TNM - Klassifikation maligner Tumoren.* In Hermanek, P., Scheibe, U., Spässl, D. & Wagner, G. (eds). Springer, Berlin Heidelberg New York Paris Tokyo.